# Nutrient-Responsive Small Signaling Peptides and Their Influence on the Root System Architecture

**DOI:** 10.3390/ijms19123927

**Published:** 2018-12-07

**Authors:** Katerina S. Lay, Hideki Takahashi

**Affiliations:** 1 Department of Biochemistry and Molecular Biology, Michigan State University, East Lansing, MI 48824, USA; laykater@msu.edu; 2 Genetics Program, Michigan State University, East Lansing, MI 48824, USA

**Keywords:** small signaling peptides, root system architecture, leucine-rich repeat receptor kinases, nutrients, nitrogen

## Abstract

The root system architecture (RSA) of plants is highly dependent on the surrounding nutrient environment. The uptake of essential nutrients triggers various signaling cascades and fluctuations in plant hormones to elicit physical changes in RSA. These pathways may involve signaling components known as small signaling peptides (SSPs), which have been implicated in a variety of plant developmental processes. This review discusses known nutrient-responsive SSPs with a focus on several subclasses that have been shown to play roles in root development. Most functionally well-characterized cases of SSP-mediated changes in RSA are found in responses to nitrogen (N) and phosphorus (P) availability, but other nutrients have also been known to affect the expression of SSP-encoding genes. These nutrient-responsive SSPs may interact downstream with leucine-rich repeat receptor kinases (LRR-RKs) to modulate hormone signaling and cellular processes impacting plant root development. SSPs responsive to multiple nutrient cues potentially act as mediators of crosstalk between the signaling pathways. Study of SSP pathways is complicated because of functional redundancy within peptide and receptor families and due to their functionality partly associated with post-translational modifications; however, as genomic research and techniques progress, novel SSP-encoding genes have been identified in many plant species. Understanding and characterizing the roles of SSPs influencing the root phenotypes will help elucidate the processes that plants use to optimize nutrient acquisition in the environment.

## 1. Introduction

Plants require macronutrients and micronutrients from the soil to grow and develop. Due to various geological processes and soil chemistry, these nutrients are heterogeneously distributed in patches and gradients [1]. Thus, plants, as sessile organisms, must use their root systems to navigate the soil profile to maximize nutrient acquisition and maintain nutrient homeostasis. The collective physical characteristics of root growth are known as the root system architecture (RSA); this term encompasses such spatial parameters as primary root length, lateral root length and density, and proliferation of root hairs [2]. RSA of most plant species is highly plastic depending on the nutrient environment [3]. These changes occur initially on a molecular level as nutrients are taken up by the plant root system, triggering signaling cascades that alter gene expression and hormone levels, which affect cell growth and differentiation of the root meristems [4]. However, there are many steps in these pathways that remain to be characterized.

One way in which plants sense changes in nutrient availability in the environment and convey the information to downstream physiological processes is through the expression of nutrient-responsive small signaling peptides (SSPs). SSPs are a class of proteins ranging from 5–75 amino acids in length. Many SSPs are derived from nonfunctional precursor proteins, which are cleaved and post-translationally-modified to mature forms which may then carry out their function as short- or long-distance mobile signals. However, other SSP families may be derived from functional precursors, the 5’ region of mRNA, primary transcripts of miRNA, or small open reading frames (sORFs) [5]. These SSP signaling pathways typically involve the secretion of peptide and perception by leucine-rich repeat receptor kinases (LRR-RKs), although some SSPs have been hypothesized to act in a receptor-independent manner, such as antimicrobial peptides [6,7]. These LRR-RKs are composed of a family of transmembrane proteins containing an extracellular LRR domain; perception of the SSP ligand occurs through binding with this domain to generate further downstream signals that may regulate growth and developmental processes. While LRR-RKs are numerous in Arabidopsis (>200 predicted members), most that are known to interact with SSPs are members of the Type XI clade [8,9]. Yet, there is a high numerical disparity between the thousands of putative SSP-encoding genes and the hundreds of LRR-RKs, and many of these pairings remain to be identified or characterized in relationship to plant development.

Regardless of the current lack of receptor identification, SSPs have been implicated in a broad range of downstream processes throughout the plant, such as meristem maintenance, cell proliferation and expansion, reproduction, and response to pathogens [6,10,11,12,13]. In the root specifically, SSPs have roles in lateral root development, nodulation, and root hair growth [14,15,16,17]. These processes may be linked to the physiological responses of plants to the soil nutrient environment as SSP-encoding gene expression is modulated in roots by changes in the nutrient availability.

**Figure 1:**
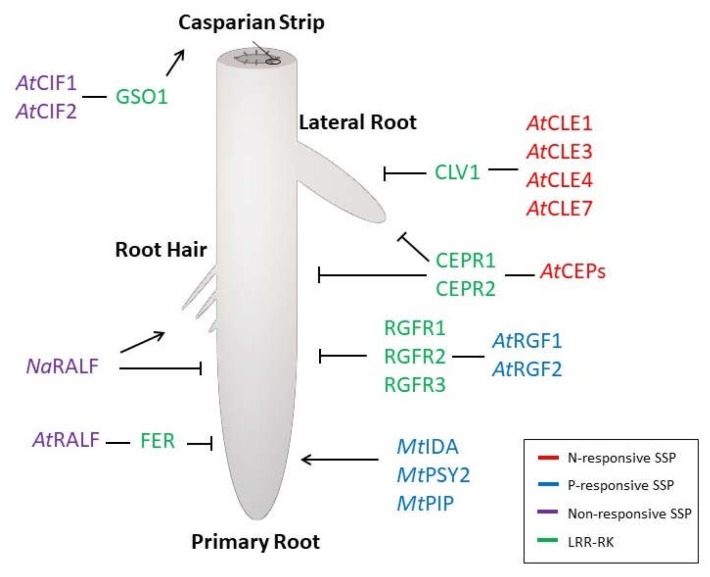
Small signaling peptides (SSP) signaling modules influencing root system architecture (RSA) and nutrient response, uptake, and homeostasis. Arrows and bars indicate promotion or repression of growth. Red: SSPs that are responsive to N-availability at the transcriptional level. Blue: SSPs responsive to P, at the transcriptional level. Purple: SSPs non-responsive to N or P but influencing the nutrient uptake or homeostasis. Green: LRR-RKs.

This review examines how nutrient signals are interpreted by SSP pathways to alter root morphology for the improvement of nutrient uptake. Most current evidence suggests that a few SSP families play important roles in regulating macronutrient-responsive changes in RSA, particularly in regard to N- or P-availability or maintaining nutrient uptake and homeostasis (Figure 1). However, recent genomic experiments have identified multiple SSP families that are responsive to nutrient cues. Additionally, certain SSPs are found to be responsive to more than one type of nutrient, suggesting they may represent signals allowing potential crosstalk between the pathways. Lastly, current challenges and novel approaches in the field of SSP research will be addressed.

## 2. Nitrogen-Responsive SSPs

Variation in availability of the essential macronutrient nitrogen (N) is known to elicit significant changes in plant RSA. Within the soil profile, nitrate (NO_3_^−^) is highly mobile and easily leaches due to the flow of water; in certain plant species, this may encourage the root system that is “steep, cheap, and deep” to acquire NO_3_^−^ that has stratified in the lower soil levels [18]. Moderate reduction in N-availability leads to an increase in lateral root length as plants forage for available N resources, while severe N-starvation inhibits the growth of primary and lateral roots in favor of survival strategies [3]. Additionally, alternate N sources, such as ammonium (NH_4_^+^) can elicit different changes in RSA. When N-depleted plants grow into a NO_3_^−^-rich patch, lateral root elongation is stimulated in order to acquire more N, but in NH_4_^+^-rich patches, more lateral roots are initiated and existing roots become more branched [19,20]. Under homogenously excess NO_3_^−^ conditions, the RSA profile exhibits long primary roots with short lateral roots, while excess NH_4_^+^ inhibits primary root growth [1]. NO_3_^−^ supply after a period of N-starvation also induces root hair development [21]. Molecular characterization of how NO_3_^−^ modulates RSA has been studied in depth and involves complex interactions between the NITRATE TRANSPORTER 1.1 (NRT1.1) and auxin signaling pathways [4]. The N-responsive SSP pathways, described below, may interact with or be part of these pre-existing processes, but direct evidence for their mechanistic interactions has not yet been found.

N-availability alters the expression of various SSPs linked to these observed changes in RSA. One peptide family in which select members have exhibited N-responsiveness is the CLAVATA3 EMBRYO SURROUNDING REGION RELATED (CLE) family. CLEs are 12–13 amino acid peptides that control plant development at various stages. While there are thirty-two CLE family members in Arabidopsis, more have been identified in leguminous species where CLE peptides are known to be involved in the autoregulation of nodulation (AON) in addition to RSA response [22,23]. The CLE family has been extensively characterized in relation to its involvement in the regulation of shoot and root apical meristem differentiation through CLAVATA3 (CLV3)-CLAVATA1 (CLV1)- and CLE40-ARABIDOPSIS CRINKLY 4 (ACR4)-mediated signaling, respectively [24,25]. Other members, however, have been shown to influence the root architecture in an N-dependent manner. Under severe N-starvation, *CLE3* (in addition to *CLE1*, *CLE4*, and *CLE7*) gene expression is induced, and mature CLE3 peptides are produced and secreted from the root pericycle, which bind to the CLV1 LRR-RLK expressed in the phloem companion cells. This interaction signals to yet uncharacterized downstream components to repress the lateral root development. This model was suggested from an experiment validating the ligand-receptor relationship in transgenic Arabidopsis lines that overexpress *CLE3*, which have a decreased lateral root density correlating with the increased *CLE3* transcript accumulation when *CLV1* is present [14]. Additionally, while the *CLE3* peptide-encoding gene is induced by NO_3_^−^-starvation, it is induced by NH_4_^+^ supply after N-starvation [26], indicating these peptides may fine tune the response in the RSA to the type of N source available.

Another SSP family with members exhibiting responsiveness to N is the C-TERMINALLY ENCODED PEPTIDE (CEP) family. The fifteen members of this family of Arabidopsis are processed to mature 15-amino-acid peptides hydroxylated at the proline residues [27]. Overexpression of multiple *CEP* genes leads to the repression of primary root growth and lateral root initiation, while the knockout lines of *CEP3* generate larger root systems with a higher lateral root density under N-limited conditions [28]. The *CEP* gene expression increases in roots undergoing local N-starvation, and mature CEP peptides translocate to the shoot as long-distance signals to interact with the CEPR1 and CEPR2 LRR-RKs, which induces a shoot-derived signal mediated by the CEP DOWNSTREAM1 (CEPD1) and CEPD2 polypeptides [29]. This signaling cascade results in the upregulation of nitrate transporters (NRT2.1) in roots exposed to relatively N-sufficient or N-rich environments to compensate for long-distance starvation [30]. Despite potential functional redundancy, individual CEP peptides may still be involved in the more specified processes governing RSA. CEP5, which putatively binds to CEPR1 to repress root growth, is negatively regulated by auxin, during lateral root initiation [31]. In *Medicago truncatula*, low nitrogen upregulates *MtCEP1*, which interacts with the CEPR1 homolog COMPACT ROOT ARCHITECTURE 2 (MtCRA2) to inhibit the lateral root growth [32,33]. Additionally, like members of the CLE family, CEP peptide families play important roles in other aspects of N-dependent root morphology in leguminous plant species, specifically in the regulation of root nodulation [34].

## 3. Phosphorus-Responsive SSPs

Phosphorus (P) is another essential macronutrient known to impact RSA. P is relatively immobile in the soil and is typically localized in higher concentrations in the topsoil layer [35]. P-deficiency, in fact, causes shorter primary roots and denser lateral roots and root hairs in Arabidopsis, likely to promote a more advantageous RSA profile for enhanced P-uptake [36]. Several additional molecular studies indicate that RSA modulation is linked to changes in P-availability. One major pathway involves the interaction between the LOW PHOSPHATE ROOT1 (LPR1) ferroxidase and the PHOSPHATE DEFICIENCY RESPONSE 2 (PDR2) ATPase—two proteins expressed in the root apical meristem that regulate the primary root growth inhibition due to P-starvation [37]. The S-DOMAIN RECEPTOR KINASE 1-6 (SDK6) and AtPUB9 proteins are also involved in P-responsive lateral root development, while ETHYLENE INSENSITIVE 3 (EIN3) directly binds to ROOT HAIR DEFECTIVE 6-like 4 (RSL4), to regulate P-responsive root hair development [38,39]. While much is known about the genetic control of these pathways in response to P-availability, it is not yet known how P-responsive SSP signaling is directly related to these studied pathways.

One of the best characterized SSP families responsive to P-availability is the ROOT GROWTH FACTOR/GOLVEN/CLE-LIKE (RGF/GLV/CLEL) family. *RGF1*, *RGF2*, and *RGF3* are induced in the meristematic cortex and epidermis of the root tip under inorganic phosphate (Pi) deprivation [40]. These three peptides were previously established to act redundantly to control root longitudinal growth [41]. A more recent study shows RGF2 and RGF1 independently alter different aspects of the primary root growth. Mutants in *RGF2* exhibit short primary roots and a hypersensitivity to low P-environments, while RGF1 was shown to control circumferential root growth through the repression of radial cellular divisions in response to a Pi deficiency. These changes are proposed to occur through the RGF-mediated signaling pathways, acting on manipulating gradients of PLETHORA (PLT) transcription factors along the root [41]. Through an extensive search for peptide receptors by photoaffinity labeling experiments, three LRR-RKs (RGFR1, RGFR2, and RGFR3) have been shown to interact with the RGF peptides to maintain the root meristem [42]. RGF-peptide signaling may directly modulate RSA in response to P-availability, but these pathways have not yet been linked to previously established P-responsive signaling pathways, such as the LPR1-PDR2-mediated pathway that inhibits primary root length under P-deficiency [4,37].

While the RGF/GLV/CLEL family regulates RSA in response to P-availability in Arabidopsis, other families have been suggested to play a role in the response based on experiments in *M. truncatula*. Members of the INFLORESCENCE DEFICIENT IN ABCISSION (*Mt*IDA), PLANT PEPTIDE CONTAINING SULFATED TYROSINE (*Mt*PSY), and PAMP-INDUCED SECRETED PEPTIDE (*Mt*PIP) families are upregulated upon P-deficiency, but exogenous application of synthetic *Mt*IDA18, *Mt*PSY2, and 9 *Mt*PIP peptides enhances the total root length, especially of the primary root [43]. It is, therefore, possible that SSP signaling pathways responding to P-stress may elicit contrary effects on RSA in different plant species. Direct receptors for these peptides have not yet been characterized in *M. truncatula*.

## 4. SSP-Dependent Changes on RSA Have Impact on Nutrient Homeostasis

SSP pathways governing root developmental processes may still influence the plant’s ability to acquire various nutrients, even if the expression of the SSP-encoding gene is not responsive itself to nutrient availability. The function of the CASPARIAN STRIP INTEGRITY FACTOR (CIF) sulfated peptides represents an example of such constitutive actions of SSPs [44,45]. CIF1 and CIF2, which are expressed in the root stele, interact with the endodermis-localized GASSHO1 (GSO1)/SCHENGEN3 LRR-RK to maintain the formation of the Casparian strip, a structure composed of suberin in the root endodermis that prevents water and nutrients from freely entering the vasculature. Interactions between these peptides and the receptor were identified using photoaffinity labeling to probe binding with members of the Type XI LRR-RK clade. Loss of CIF-GSO1 signaling impacts the homeostasis of potassium (K), as well as the micronutrients zinc and magnesium, and can also increase shoot sensitivity to an iron-excess, as the loss of Casparian strip integrity may lead to ion leakage between the xylem and the soil [46]. Alternatively, SSP signaling can promote RSA changes that improve nutrient uptake. Expression of the RAPID ALKALIZATION FACTOR (RALF) peptides in the root is linked to a shorter primary root growth and increased cytoplasmic calcium levels. In Arabidopsis, these RALF peptides interact with the FERONIA (FER) LRR-RK to repress cell elongation in the root; however, in *Nicotiana attenuata*, NaRALF1 additionally promotes root hair formation, which is proposed to improve P-uptake [47,48]. These examples indicate that SSP signaling modules involved in RSA modulation potentially affect the ability of plants to take up essential nutrients, even if the SSP-encoding gene does not exhibit responsiveness to that specific nutrient cue.

Other non-nutrient responsive SSP pathways are known to impact and modulate RSA, which may indirectly affect nutrient uptake. Many of these established pathways have been shown to have downstream effects on cell structure and maintenance. Among the members of the CLE family, CLE40-ACR signaling is integral for the maintenance of the root apical meristem [25]. IDA peptides in Arabidopsis signal to the HAESA (HAE) and HAESA-LIKE2 LRR-RKs to promote lateral root emergence through an enhanced cell separation [15]. PHYTOSULFOKINE (PSK) also interacts with its receptor PSKR to enhance root growth through cell elongation [49]. While these developmental modules have not yet been studied in relation to nutrient cues, modulation of these RSA phenotypes may aid in the uptake of certain nutrients from the heterogeneous soil environment.

## 5. Transcriptomic Studies Reveal Potential SSP Candidates for Functional Characterization

Only a small percentage of SSPs have been functionally characterized for their involvement in nutrient response and RSA. Potential candidates for further research are largely identified after their nutrient responsiveness are exhibited at the transcriptional level. Relevance of SSPs to regulatory pathways responding to nutritional availability and their potential influence on root development can be mined from the transcriptome datasets examining the effect of nutrient supply on gene expression or RSA. Microarray data of Arabidopsis grown on split-root media, with half of the root system exposed to N-replete environments and the other half in N-deficient conditions, shows a differential expression of thirty-one SSPs (including various CLEs and CEPs) from multiple SSP families [50,51]. These differentially expressed SSPs are proposed to be responsive to long-distance N-signals or be active as mobile signals for communication between root segments. These pathways could contribute to the downstream changes in RSA observed between split root conditions, namely, the compensatory root growth observed in nitrate-rich environments when distal root segments experience N-limitation [50]. Additionally, nitrate supplementation after N-starvation has been shown to induce root hair formation in a process mediated by TGA1 and TGA4 transcription factors [21]. Among potential TGA1/TGA4 downstream targets are CAP (Cysteine-rich secretory proteins, Antigen 5, and Pathogenesis-related 1 protein) (CAPE) peptides, which belong to an SSP family initially investigated for its role in pathogen response and salt tolerance [52,53]. These CAPEs are differentially expressed after NO_3_^−^ supply, but this response is ablated in the *tga1/tga4* double knockout; however, the direct effect of CAPE expression on root hair phenotype, once again, remains to be observed or functionally characterized.

N and P are indeed the well-characterized macronutrients affecting the SSP-mediated changes in RSA, likely because they induce the most dramatic visible changes in root morphology. However, other macronutrients, such as sulfur (S) and K, as well as various micronutrients may also act through SSP pathways to influence growth and metabolism in the root. Analysis of *M. truncatula* transcriptome after S- and K-deprivation showed that seventy-two SSP-encoding genes were differentially regulated by S, and one (*Mt*Legin13) was downregulated by a K-deficiency [43]. Additional research must be conducted on these SSPs to identify the downstream processes and potential effects on the RSA.

## 6. Gene Expression Reveals Potential for Nutrient-Dependent Crosstalk Involving SSP Pathways

One further challenge to interpret the impact of nutrients on the RSA is that most of the experiments reported in the literature only examine the effect of a single nutrient. Plants growing in soil, however, experience combinatorial nutrient cues that could independently cause contradictory changes in RSA. The moderate effects of certain nutrients, such as S and K, on RSA may also be magnified when combined with fluctuations in other nutrient availabilities [54]. Crosstalk mechanisms between the molecular pathways likely exist to fine-tune RSA in response to these complex environments. These mechanisms may involve SSPs, as certain SSP-encoding genes have been shown to respond to multiple nutrient deficiencies.

These cues may be derived from different functional forms of the same nutrient. For example, *CLE3* expression is repressed by nitrate, the more desirable source of N used by the plant, but highly induced by ammonium [26,55]. This can potentially be used as a sensing mechanism for an N-context in the surrounding environment and lead to less lateral roots emerging in the ammonium-rich areas and a higher proliferation in the comparatively nitrate-rich patches to maximize the N-uptake efficiency. Other SSPs may still act as integrators for diverse nutrient cues. CLE2, which is also implicated in the lateral root development through interactions with CLV1, is responsive to not only NO_3_^−^ but also to sulfate and phosphate, while other CLE family members exhibit responsiveness to glucose and iron availabilities [56]. Corroborating with these results suggesting potential multifactorial nutrient responses of CLEs in Arabidopsis, multiple CLE homologs (*Lj*CLEs) in the *Lotus japonicus* have been shown to be upregulated in response to P-supply, although these have not been linked to any RSA changes [57]. These trends are not specific to Arabidopsis and have recently been examined in a large-scale study exposing *M. truncatula* to N, P, K, and S starvation and resupply. Two hundred forty nutrient-responsive SSPs were purportedly involved in the receptor-mediated signaling; while 61% of these genes were responsive to a single macronutrient deprivation in the roots, the remainder, including the *Mt*CLE family members *Mt*CLE05 and *Mt*CLE34, were responsive to two or three conditions [43]. Research remains limited on the effect of multiple nutrient cues acting through individual SSP pathways.

## 7. Conclusions

The field of SSP research is greatly expanding. In Arabidopsis, over a thousand peptide encoding genes have been identified [58]. Likewise, in the *M. truncatula*, recent genome re-annotations have identified 1970 homologs of known SSP gene families and 2455 potentially novel SSP-encoding genes [43]. Increasing study of SSPs has led to the development of the PlantSSP database (http://bioinformatics.psb.ugent.be/webtools/PlantSSP/, accessed on 8 November 2018), which predicts close to 40,000 SSPs and over 4000 SSP families based on the annotations of 32 plant species, many of which have agricultural relevance [59]. Despite such an expansion of information through in silico re-annotations, knowledge of how these nutrient-responsive SSPs influence RSA remains limited, particularly in these crop species, due to the lack of phenotype correlation and identification of receptors or downstream signaling targets, among other challenges. Still, many strategies have been employed to expand the SSP signaling research and establish connections between the nutrient environment and root physiology.

Many experiments performed to study SSPs have relied on forward and reverse genetics using transgenic overexpression lines of peptide-encoding genes or knockout mutant lines. One study has also suggested the use of antagonistic peptides to artificially generate a dominant-negative effect to examine the loss of peptide activity [60]. However, these tools are complicated by a functional redundancy within the peptide families [14,41]. Some peptides, such as CLE1 and CLE4, may even have identical mature peptide sequences and overlap in tissue localization [61]. Single mutant studies may not be efficient enough to fully characterize the roles of redundant SSP families; loss of function mutants created using T-DNA insertions are difficult to generate due to the small size of SSP-encoding genes, and alternative strategies, such as generating antagonistic peptides, may still not result in detectable RSA phenotypes. Emergent technologies, such as gene editing, may aid in studying these complex pathways. CRISPR/Cas9 has been used to generate individual knockouts in CLE-peptide encoding genes [62]. The high specificity of this gene editing system was shown to be effective for generating mutants in this family, which has been difficult in the past due to the small gene size of the CLE family members. While many of the mutants generated using this approach still did not have detectable developmental phenotypes because of the redundancy of peptide sequences, CRISPR/Cas9 has also been shown to be effective in generating higher order mutants within peptide families. This method has been used to generate multiplex knockout lines of the 6 RGF/GLV/CLEL family members; while the stable mutant has not yet been characterized phenotypically, the method was shown to be highly specific with no off-target effects [63].

Another specific consideration is that many experiments rely on the use of exogenously applied synthetic peptides. While these may indeed phenocopy the transgenic overexpression lines and provide a relatively quicker means of analysis, many synthetically-generated peptides lack post-translational modifications that are necessary for proper biological functioning of the SSPs [9]. Conserved post-translational modifications within many Arabidopsis SSP families have been identified using the structural analysis methods, such as nuclear magnetic resonance (NMR) and LC–MS/MS [64,65]. Additionally, the concentration at which they are applied to the medium greatly exceeds the physiological levels, which are estimated to be in the nanomolar range [60].

Lastly, challenges are present not only in studying SSPs themselves, but also in determining associated LRR-RKs. Many studies on the downstream developmental effects of SSP pathways rely on using the mutant lines of proposed receptors. However, SSPs may bind to multiple receptors and individual receptors may also interact with multiple SSPs [42,44,45]. Furthermore, receptors may be functionally redundant, which can complicate the understanding of how the SSP pathways impact plant development. For example, the CLV3 peptide can be perceived by both CLV1 and the BARELY ANY MERISTEM (BAM) kinase to regulate stem-cell specification; the *clv1 bam* double mutant results in a much more severe phenotype than the single mutants in *clv1* or *bam* [66]. This redundancy in the receptor kinase function is also observed with the CLV2-CORYNE (CRN) complex and the RECEPTOR-LIKE PROTEIN KINASE 2 (RPK2) [67,68,69,70], and in the root apical meristem, where CLE40 can bind to both CLV1 and ACR4 [71]. Aside from issues of redundancy, LRR-RKs may form hetero- and homo-dimeric protein complexes with leucine-rich repeat (LRR) proteins, associated with additional kinases [67,68,69,70,71,72], or with coreceptors, such as somatic embryogenesis receptor-like kinases (SERKs), further complicating the identification and study of peptide-receptor pairings [73,74].

While there remain significant technical challenges, SSP signaling modules identified recently have shown a great promise as regulators of root development, controlled in response to nutrient environmental cues. SSP signaling and biology can be applied to various agricultural practices. Improving the basic understanding of these pathways and how they mediate nutrient-responsive changes in the RSA in model organisms such as Arabidopsis and *M. truncatula* may be further translated in crops or other leguminous species. More directly, application of validated synthetic peptides to seeds or plants growing in nutrient-limited environments is proposed as a non-transgenic means to improve plant growth and yield. Current research in the field has identified the key roles of SSPs in N- and P-responsive root development, as well as other SSPs either responsive to other nutrients or influential in nutrient uptake and homeostasis. Large-scale genomic datasets have also introduced a better understanding of the number and variation in the putative SSP families, which could potentially impact the RSA or be involved in the crosstalk of various nutrient cues. Further research advancement in this area to elucidate the roles of potentially hundreds of nutrient-responsive SSPs is imperative to fully understand the integration of nutrient cues in RSA phenotypes.

## Abbreviations

RSA	Root system architecture
SSP	Small signaling peptide
LRR-RK	Leucine-rich repeat receptor kinase
sORF	Small open reading frames
NRT	Nitrate transporter
CLE	Clavata3/embryo surrounding region related
AON	Autoregulation of nodulation
CLV	CLAVATA
CEP	C-terminally encoded peptide
RGF/GLV/CLEL	Root growth factor/Golven/CLE-like
LPR	Low phosphate root
PDR	Phosphate deficiency response
PLT	PLETHORA
IDA	Inflorescence deficient in abscission
PSY	Plant peptide containing sulfated tyrosine
PIP	PAMP-induced secreted peptide
CIF	Casparian strip integrity factor
GSO	GASSHO1/SCHENGEN3
RALF	Rapid alkalization factor
FER	FERONIA
HAE	HAESA
PSK	PHYTOSULFOKINE
CAPE	Cysteine-rich secretory proteins, antigen 5, and pathogenesis-related 1 protein
NMR	Nuclear magnetic resonance
LC-MS/MS	Liquid chromatography with tandem mass spectrometry
BAM	Barely any meristem
CRN	CORYNE
RPK	Receptor-like protein kinase
SERK	Somatic embryogenesis receptor-like kinases
